# Zero party data between hype and hope

**DOI:** 10.3389/fdata.2022.943372

**Published:** 2022-08-30

**Authors:** Andrea Polonioli

**Affiliations:** Coveo Solutions Inc, Quebec City, QC, Canada

**Keywords:** personalization, Ecommerce, zero party data, privacy, big data, retail, consumer behavior

## Abstract

Zero Party Data (ZPD) is a hot topic in the context of privacy-aware personalization, as the exponential growth of consumer data collected by retailers has made safeguarding data privacy a key priority. Articles arguing for the value of ZPD to improve personalization and engender consumer trust have appeared in the popular press, in business magazines as well as in academic journals. Advocates of ZDP argue that instead of inferring what customers want, retailers can simply ask them. Provided that the value exchange is clear, customers will willingly share data such as purchase intentions and preferences to improve personalization and help retailers create a picture of who they are. While the rise of ZPD is a welcome development, this paper takes issue with the claim that ZPD is necessarily accurate as it comes directly from the customer. This view is at odds with established conclusions from decades of research in the social and cognitive sciences, showing that self reports can be influenced by the instrument and that people have limited insight into the factors underlying their behavior. This paper argues that while ZDP disclosures are an important tool for retailers, it is critical to carefully understand their limitations as well. The paper also provides a catalog of biases for identifying potential problems in survey design to help practitioners collect more accurate data.

## Introduction

Retailers leverage a number of marketing strategies that use consumer data to influence purchase decisions (Gerrikagoitia et al., 2015; Fisher and Raman, 2018). A significant trend among retailers has been to use personalization technologies to gather data about customers' online behavior in order to tailor experiences and offerings to increase their relevance and meet customer preferences (Kalaignanam et al., 2018). Fuelled by the recent growth of consumer data collected by retailers across multiple touchpoints (Santoro et al., 2019), data-driven personalization strategies have quickly become the “life-blood of retail” (National Retail Federation, 2013).

Recently, however, the importance of safeguarding data privacy in the context of personalization has become increasingly apparent due to changes in the regulatory and technological landscape as well as evolving consumer perceptions.

First, regulations such as the General Data Protection Regulation (GDPR) and the California Consumer Privacy Act (CCPA) are in place precisely to strictly govern companies in their usage of customer data. Gartner estimates that by 2023, 65% of the world's consumers will have their data protected by regulations (Gartner, 2020).

Second, third-party cookies are expected to be fully deprecated from all web browsers in mid-2024, when Google Chrome will join Apple's Safari and other browsers in banning third-party cookies that help companies understand consumer behavior across the internet (Wiggers, 2022).

Third, consumers have become increasingly concerned about how retailers manage their data (Lopez et al., 2016; Bandara et al., 2020). While greater personalization typically increases service relevance and customer adoption, it may also increase customers' sense of vulnerability. Consumers do not want their privacy to be abused and demand that their personal information remains safe, secure, and leveraged uniquely for benign purposes (Kutty et al., 2021).

Recent attempts to navigate the tensions between relevance, technology and privacy have produced a substantial body of literature and continue to motivate scholars and practitioners working in this space (Elizabeth et al., 2016; Karwatzki et al., 2017). Interestingly, a proposal that was recently made is to reimagine consumer information exchanges by using so-called zero-party data (ZPD) disclosures (Martin and Palmatier, 2020; Schmidt et al., 2020; Hall, 2021; Krafft et al., 2021; Mishra, 2021; Quach et al., 2022). ZPD refers to information that consumers knowingly and willingly provide to retailers in exchange for more meaningful personalization. Simply put, instead of inferring what customers want or need, retailers can simply ask customers to share their personal data, such as purchase intentions and preferences. The promise is that if companies offer a clear value exchange, consumers will volunteer their data, thus improving personalization and helping create a picture of who they are.

The rise of ZPD is a welcome development. However, this paper also draws attention to a fundamental yet surprisingly overlooked limitation of ZPD collection practices. Specifically, a distinctive feature of ZPD collection practices is that they rely on questionnaires, polls, and quizzes. The excitement around ZPD disclosures is to a large extent based on the assumption that self-reports are transparent, reliable, and provide access to highly accurate data. However, there are problems with the claim that ZPD is necessarily accurate as it comes directly from the customer. As it turns out, this view is at odds with established conclusions from decades of research in the social and cognitive sciences, showing that self-reports can be influenced by the instrument and that people have limited insight into the factors underlying their behavior (Tourangeau and Rips, 2000; Dunning et al., 2004; Wilson, 2004; Choi and Pak, 2005; Sedgwick, 2013; Schaeffer and Dykema, 2020). Hence, the paper argues that ZPD should not be seen as a silver bullet in the quest for privacy-aware personalization. Instead, ZDP disclosures should best be seen as a new arrow in retailers' quiver and intended to complement rather than replace first-party data disclosures.

The paper is structured as follows. Section Zero party data: Definition, use cases and rationale surveys the recent literature on ZPD and clarifies its relevance to retailers, its main use cases, and the rationale for the surge of interest in ZPD. Section Limitations of ZPD collection practices puts forward an argument to the effect that ZPD collection practices suffer from non-trivial limitations. Section Conclusion delivers the conclusion.

## Zero party data: Definition, use cases and rationale

The goal of this section is three-fold. First, it defines what zero-party data (ZPD) is. Second, it clarifies the main use cases for ZPD collection. Third, it discusses the rationale for the growing interest in and adoption of ZPD.

### Defining zero party data

The term zero-party data (ZPD) was recently popularized by industry analysts from Forrester Research. More precisely, Khatibloo and colleagues defined zero-party data (ZPD) as “data that a customer intentionally and proactively shares with a brand. It can include preference center data, purchase intentions, personal context, and how the individual wants the brand to recognize her” (Khatibloo et al., 2017).

A couple of remarks are in order. First, while it has become common practice to credit analysts from Forrester Research with the introduction of the notion of ZPD, it should be noted that earlier uses of the term ZPD can be observed in the literature, although in such cases the notion was used with a different meaning. In particular, Budak et al. (2016) claim that ZPD “encompasses instances in which data on a user's past actions are not directly involved in prompting the shopping session.” This is not, however, what the burgeoning research on ZPD refers to when using the concept of ZPD, which is instead used to refer to data that customers proactively and deliberately share in exchange for personalized experience (Britt, 2020; Gilliland, 2020; Yun et al., 2020).

Second, while the notion of ZPD has gained increasing popularity over the past years, other terms are occasionally preferred by scholars working in the field privacy-aware personalization. For instance, Hartemo (2021) clarifies that they “refer instead to “volunteered data” to cover voluntarily given zero-party-data.” However, instead of avoiding the use of the term ZPD, which is now widely used by both academics and practitioners, we prefer to clarify and further refine the meaning of ZPD. Specifically, while the notion of ZPD may still appear to be a partly unclear construct to some, it is possible to fruitfully leverage some widely established notions and characterizations from the literature on recommender systems (Jawaheer et al., 2010). In particular, this paper favors a working definition of ZDP that is framed in terms of *explicit feedback*, which supposedly allows consumers to unequivocally express information about themselves and the experiences that they are receiving. This includes preferences, which are always a comparative evaluation of a set of items, but also expressions of liking of a particular experience, as well as information about goals and intent.

By virtue of this characterization, it is easy to appreciate the difference between first party data and ZPD. As pointed out by Khatibloo et al. (2017), while brands might historically have considered ZPD data to be “first party,” consumer expectations have forced the need for a new term and a new way of treating this kind of personal information. Consensus has in fact emerged that it is indeeed helpful to introduce a distinct class of data. It seems fair to conclude that there are two features that are especially salient in the context of ZPD. First, ZDP is shared *intentionally* by customers. This can reflect the fact that the customer is expressing trust and a willingness to provide personal information, or simply that she is expecting a more valuable experience. Second, in the context of ZPD, feedback, preferences and intent are *not inferred* but rather *shared* by customers.

This is in contrast with approaches to personalization based on first-party data and in-session behavior (Yu et al., 2020). More precisely, in the context of Ecommerce personalization, first-party data is generally best characterized as behavior-based data associated with a tracking cookie. Such first-party, clickstream data can be rich and effective (Iwanaga et al., 2019; Requena et al., 2020; Zavali et al., 2021) and in an Ecommerce setting this can include, for example, data about the search terms that customers use, which pages they visit first, what they click on and how much time they spend on a certain page but also which items they browse, add to cart and purchase. This data contains the trajectory of (prospective) clients on a company's website and provides very detailed records of what visitors do when navigating an e-store. However, in the case of first-party data, preferences and intent are *inferred* rather than *disclosed intentionally* by consumers. Put simply, while first-party data is rich with behavioral data and implied interest, ZPD provides explicit interest and preferences.

Further, based on the above-mentioned definition of ZPD, distinguishing between ZPD and third party data is rather straightforward. Third-party data can provide useful demographic and psychographic attributes about customers and prospects. However, unlike third-party data, which is collected by aggregators through third-party cookies and various tracking techniques (Lotame, 2018), ZPD is collected voluntarily and directly from customers. ZPD is hence quite different: instead of inferring what customers want from a set of aggregated data, it provides explicit intention and preference data for each user. And, in an after-GDPR era, it passes the consent test in a way that third-party data does not.

### Zero party data use cases

Most of the interest in ZPD is due to its application and role in the context of Ecommerce personalization, where it can arguably help better capture intent and preferences of anonymous as well as of repeat customers (Britt, 2020; Gilliland, 2020; Yun et al., 2020).

Importantly, however, ZPD is relevant to several use cases, and not just for better and privacy-aware personalization. In what follows, we review some prominent use cases for ZPD collection.

#### Market research

ZPD can provide brands with an opportunity to solicit customer feedback and ensure their products and services are resonating with customers. For instance, brands and retailers can use ZPD to scope out potential partnerships or product development opportunities. NARS and North Carolina Education Lottery used ZPD to scope out potential partnerships (Prasad, 2022).

#### Communication preferences

ZPD can provide users with options to choose from, so they can specify what communication they wish to receive from a brand or retailer. Consumers can offer exact information on where, when, and how often they want to receive messages.

#### Customer feedback

ZPD can give brands an opportunity to understand whether customers are satisfied with the experiences they received. This use case has become rather popular on websites and applications. From YouTube to Quora, from Facebook to Linkedin, requests to submit explicit feedback have become increasingly common.

#### Profile curation

Players such as Amazon allow customers to not only track every product that they have ever searched and viewed since the time of the creation of their account, but also to review and curate their history to make sure that experiences are relevant. Curation and item removal to improve the accuracy of a customer profile can be understood as a relevant ZPD use case. Arguably, this use case is mostly relevant to a select group of Ecommerce players, as most retail websites only have a limited number of recurring customers who have an account and log in Tagliabue et al. (2021).

#### Site navigation

Navigating a website as a first-time visitor can be confusing. Yet, ZPD can be used for self-segmentation and site navigation. Business Development Bank of Canada uses for instance a ZPD widget to quickly direct site visitors to the content or products that align with their financial goals, even when the bank has no idea who the visitor is (Forrester, 2021). This ensures that new visitors find relevant content without needing to share personal financial details with a bank they don't already have a relationship with.

Since explicit feedback and ZPD come in different ways, it should come as no surprise that there can also be different tools that can be used to collect data. For instance, it can be easy to implement a functionality into a website that allows users to evaluate the content served and experiences received by submitting customer feedback. Preference centers are a great tool for capturing ZPD, but they work best for existing customers as they usually require a login and centralize all of an individual's stated preferences and subscriptions.

### The case for zero-party data

Retailers have started to look at ZPD as powerful tools to make their personalization strategies more effective and privacy-aware at a time in which third-party data is becoming less available and reliable and in which customers are becoming increasingly sensitive to privacy considerations and concerns. Not only does evidence reveal that consumers have concerns over privacy abuses (Kutty et al., 2021; Maseeh et al., 2021), but also shows that customer profiles built from purchased third-party data may often not be worth the cost, due to the black-box nature of how profiles are created (Neumann et al., 2019). This matters greatly, as personalization appears to be relevant to not only delivering a great customer experience but also to achieving business outcomes. Notably, besides the research conducted by consultancy firms, suggesting that personalization can drive 10 to 15 percent revenue lift (McKinsey., 2022) and that 78% of US online adults have chosen, recommended, or paid more for a brand using personalized digital experiences or services (Forrester, 2018), the potential payoff of personalization has been frequently reported also in scholarly, peer-reviewed research (Yoganarasimhan, 2020; Yu et al., 2020).

Unfortunately, however, despite the increasing adoption of ZPD collection practices in the context of privacy-aware personalization, the empirical evidence available on the causal impact and value of ZPD is scant to nil. Specifically, most of the evidence comes from marketing assets published by technology vendors that provide retailers with tools for easy collection and activation of ZPD. While many of these assets are insightful and refer to increases in Conversion Rates and other business critical KPIs, the statistics must be taken with a grain of salt. To our knowledge, the only peer reviewed study that offers a controlled experiment in support of the value of ZPD examines the effect of a random allocation of email recipients to two different conditions, one that utilized ZPD and another one that utilized observed data, finding better performance for the former (Hartemo, 2021).

While empirical evidence is limited and fragmented, plenty of articles explicitly presented under the banner of ZPD and which have appeared in the popular press and in prominent outlets point to critical benefits for consumers as well as retailers. In particular, there are two main intertwined considerations that are typically provided to argue for the value of ZPD.

#### The argument from increased transparency

First, since in the context of ZPD disclosures consumers know what information they are sharing and why, they can decide how much to reveal, which constitutes an ideal condition to empower and foster trust (Martin and Palmatier, 2020; Schmidt et al., 2020; Hall, 2021; Krafft et al., 2021; Mishra, 2021; Quach et al., 2022). Specifically, trust exists when one party has confidence in the other party's reliability and integrity, as may be assumed when the consumer is giving permission for an organization to personalize experiences and recommendations. For example, Martin and Palmatier (2020) recently argued that “*because zero party data disclosure is based on an existing consumer–retailer agreement, various transparency and control benefits should arise, which can engender trust and reduce both feelings of violation and the negative effects of unexpected data breaches*.”

#### The argument from increased accuracy

Second, a fundamental advantage attributed to ZPD comes from the increased accuracy of volunteered information. For instance, a recent article published in Forbes claimed that “*Zero-Party Data Is The New Oil*,” arguing that “*because customers supply it directly, there's less opportunity for errors or inaccuracies*” (Gozman, 2022). Another article published instead in the Entrepreneur presented ZPD as “the new secret weapon for brands (Rotter, 2022).”

This argument has been especially influential among practitioners and analysts and the excitement around ZPD disclosures is to a large extent based on the assumption that self-reports are transparent, reliable, and provide access to highly accurate data.

Yet, in the remainder of this paper it will be argued that precisely because ZPD relies heavily on conversation-like exchanges, practitioners as well as researchers working on ZPD should not overlook the critical insights received from research on the “Science of Asking Questions” (Schaeffer and Dykema, 2020) and the psychology of survey answers (Tourangeau and Rips, 2000), which highlight several potential non-trivial shortcomings and limitations that need to be carefully considered. While surveys are the most direct means to investigate ordinary reactions and continue to be widely used in many of the social sciences, they have important limitations as a source of data, meaning that ZPD should ideally be always combined with data from other approaches and that guidelines should be offered to help practitioners collect accurate data.

## Limitations of ZPD collection practices

Questionnaires can be a useful tool, but the limitations of the survey-based research method must be considered as well. Surveys to elicit ZPD are vulnerable to response biases, phrasing ambiguities, and the widely recognized finding that people have only limited conscious insight into their behavior (Wilson, 2004; Carmel, 2011). Self-reports can turn out to be deeply influenced by the research instrument and at every step of the ZPD response process, the information respondents provide depends in non-trivial ways on the specifics of the questionnaire (Schwarz and Oyserman, 2001).

### Careless responding

Optimizing the quality of survey data is critical to making reliable, robust inferences for improved personalization. A major challenge to the data quality of questionnaires for ZPD collection is careless responding, which is also referred to as insufficient effort responding (e.g., Bowling et al., 2016). Careless responding occurs when survey participants do not read or pay attention to item content, thus failing to provide accurate responses (Huang et al., 2012; Meade and Craig, 2012).

Respondents are more likely to self-report responding randomly toward the middle or end of a long survey, but even short questionnaires can prompt inaccurate responses. It is worth referring here to recent findings on users' interactions with consent notices: users tend to automatically consent without even viewing notices (Cate, 2010; Trevisan et al., 2019; Nouwens et al., 2020), since these are perceived as an obstacle to the visitors' main goal, namely accessing the service. This behavior has been explained by referring to users' struggles to understand how to make adequate decisions about their privacy preferences; yet also in cases where they are informed of the implications of the decisions, they opt for short-term benefits over long-term privacy (Nouwens et al., 2020).

### Vague questions

People communicate and understand each other using a multitude of concepts (Jaccard and Jacoby, 2010). Concepts, however, normally trigger associations in our minds, and individuals can have dissimilar associations when they are faced with the same concept. When used in surveys, vague concepts may have the effect that different individuals understand the same questions in different ways (Brady, 1985) and result in incomparability (King et al., 2004). Vague questions lead respondents to provide vague answers. For example, asking visitors to self-segment by answering the question as to whether they are seasoned snowboarders might introduce an element of vagueness. Different visitors may interpret the meaning of “seasoned” differently.

### Uncommon words

Research on survey design has shown that uncommon and difficult words should always be avoided when designing a questionnaire (Choi and Pak, 2005). Brands and retailers should therefore ensure that they use words that are common in questionnaires and acknowledge that especially non-native speakers of English are even more likely to be unfamiliar with less common words.

### Faulty scales

Forced choice (sometimes referred to as insufficient category) can also result in limited data accuracy. Questions that provide too few categories can force respondents to choose imprecisely among limited options (Foddy, 1993). For example, asking customers whether one agrees or disagrees without offering a “don't know” category may produce a bias because respondents who have no opinion are forced to select an answer that may or may not reflect their true attitude.

### Central tendency bias

Respondents usually avoid ends of scales in their answers. This central tendency bias, which inclines participants to avoid the endpoints of a response scale and to prefer responses closer to the midpoint, is widely considered “one of the most obstinate” response biases in the field of psychology (Stevens, 1971, p. 428). Users are often conservative and wish to be in the middle. For instance, respondents are more likely to check “Agree” or “Disagree” than “Strongly agree” or “Strongly disagree.”

### Accessibility bias

Accessibility biases influence judgments and decision tasks and refer to the influence of how easily information can be retrieved (cf. Iyengar, 1990; Schwarz and Vaughn, 2002). In the context of ZPD, for instance, answers to questions regarding customers' preferred brands can simply reflect how readily high awareness brands come to mind (Hauser, 2011).

### Better-than-the average effects

People are often subject to flawed self-assessments (Dunning et al., 2004). In several cases, they hold unrealistic views about their position with regard to others. Consider for instance the classic example of this tendency is a 1981 survey of automobile drivers in Sweden, in which 90% of them described themselves as above average drivers (Svenson, 1981). These effects have been shown to be widespread (Moore and Healy, 2008). This has non-trivial implications for ZPD collection practices. For instance, asking customers to choose whether they are reasonably experienced snowboarders, seasoned snowboarders, or amateurs might not lead to the most accurate and meaningful self-segmentation.

### Desirability biases

Asking website visitors questions about their brand preferences can trigger social desirability biases, whereby people project themselves in the most favorable way relative to prevailing social norms and beliefs about what is desirable (King and Bruner, 2000; Krumpal, 2013). For instance, previous work pointed out that social desirability of consumption related to socially desirable outcomes may affect consumers' responses (Costanigro et al., 2011). In the context of ZPD collection practices, asking visitors about their preferred brands can trigger answers that do not reflect consumers' real preferences but rather project them in the most favorable way by selecting brands that are perceived as highly desirable.

### Affective forecasting

Psychological research on “affective forecasting” (Wilson and Gilbert, 2005) consistently shows that people do a poor job at predicting their future preferences and emotions. This finding is important for brands and retailers, since ZPD collected through surveys can offer limited indications as to what consumers will do, expect and prefer in the future. A seminal experiment in affective forecasting by Kahneman and Snell (1992) revealed a systematic bias. At a specified time on each of seven consecutive days, participants would eat a bowl of yogurt while listening to the same piece of music. On the first day, they shared how much they liked both the yogurt and the piece of music and later predicted how much they would like them on the seventh day. While participants claimed that they would like the music and the yogurt much less, there was no consistent pattern of decline. In fact, it turned out that they liked the yogurt more on the seventh day than on the first, and their decline in appreciation for the music was smaller than expected. Further research has shown that systematic biases in “affective forecasting” are robust (Wilson and Gilbert, 2005).

## Conclusion

The rise of ZPD is a welcome development in the context of privacy-aware personalization. However, this paper threw doubt on the claim that ZPD is necessarily accurate as it comes directly from the customer. More precisely, this paper aimed at making scholars and practitioners aware of a blind spot in the fast-growing literature on ZPD. This flurry of research has surprisingly overlooked conclusions drawn from decades of research in the social and cognitive sciences to the effect that self reports can be deeply influenced by the instrument and that people have limited insight into critical factors that underlie their behavior. Besides arguing that surveys suffer from non-trivial shortcomings and that ZPD should always be complemented with other kinds of data, this paper provided a catalog of fundamental types of biases and challenges that are likely to affect the quality of the data collected, thus facilitating the identification of potential problems and serving as a resource for Ecommerce and marketing practitioners using questionnaires to retrieve ZPD.

## Data availability statement

The original contributions presented in the study are included in the article/supplementary material, further inquiries can be directed to the corresponding author.

## Author contributions

AP conceived the research and wrote the paper.

## Conflict of interest

Author AP was employed by Coveo Solutions Inc.

## Publisher's note

All claims expressed in this article are solely those of the authors and do not necessarily represent those of their affiliated organizations, or those of the publisher, the editors and the reviewers. Any product that may be evaluated in this article, or claim that may be made by its manufacturer, is not guaranteed or endorsed by the publisher.
